# Multiscale Simulation of Semi-Crystalline Polymers to Predict Mechanical Properties

**DOI:** 10.3390/polym13193233

**Published:** 2021-09-22

**Authors:** Tobias Daniel Horn, Dario Heidrich, Hans Wulf, Michael Gehde, Jörn Ihlemann

**Affiliations:** 1 Department of Solid Mechanics, Chemnitz University of Technology, 09126 Chemnitz, Germany; hans.wulf@mb.tu-chemnitz.de (H.W.); joern.ihlemann@mb.tu-chemnitz.de (J.I.); 2 Department of Plastics Engineering, Chemnitz University of Technology, 09126 Chemnitz, Germany; dario.heidrich@mb.tu-chemnitz.de (D.H.); michael.gehde@mb.tu-chemnitz.de (M.G.)

**Keywords:** semi-crystalline polymer, multiscale simulation, lamella, spherulite, homogenization, cellular automaton

## Abstract

A multiscale simulation method for the determination of mechanical properties of semi-crystalline polymers is presented. First, a four-phase model of crystallization of semi-crystalline polymers is introduced, which is based on the crystallization model of Strobl. From this, a simulation on the nanoscale is derived, which models the formation of lamellae and spherulites during the cooling of the polymer by using a cellular automaton. In the solidified state, mechanical properties are assigned to the formed phases and thus the mechanical behavior of the nanoscale is determined by a finite element (FE) simulation. At this scale, simulations can only be performed up to a simulation range of a few square micrometers. Therefore, the dependence of the mechanical properties on the degree of crystallization is determined by means of homogenization. At the microscale, the cooling of the polymer is simulated by a cellular automaton according to evolution equations. In combination with the mechanical properties determined by homogenization, the mechanical behavior of a macroscopic component can be predicted.

## 1. Introduction

The mechanical properties of a semi-crystalline polymer are significantly influenced by the microstructure that is formed during cooling from the melt. In order to predict the material properties of the semi-crystalline polymer, it is important to accurately predict the formation of the microstructure and to correctly derive the relationship between the microstructure and the mechanical properties.

The macromolecules of a semi-crystalline polymer form a lamellar structure when cooled from quiescent melt. The perception of this structure was decisively influenced by Keller [1]. In case of sufficiently slow cooling from quiescent melt, these crystalline lamellae form superstructures called spherulites. In the literature, the corresponding formation of the microstructure is simulated with the focus on the scale of spherulites. So, Charbon and Swaminarayan simulated a spherulite formation in a temperature field using a front tracking method [2]. Raabe [3] has used a cellular automaton to calculate the growth of spherulites in a polymer melt under quiescent conditions. Later, this model was extended to consider weak shearing as well [4]. Michaeli et al. [5] have modeled the solidification of semi-crystalline polymer and thermally induced quiescent nucleation was taken into account. Their model was extended in order to consider flow-induced nucleation [6].

Another long-standing challenge is the prediction of the corresponding material properties of semi-crystalline polymers. The main focus has been on the development of mean field micro-mechanical models, where the semi-crystalline structure is idealized in different ways. Van Dommelen et al. [7] modeled the microstructure as an aggregate of two-phase layered composite inclusions. Uchida et al. [8] have predicted the elasto-viscoplastic behavior by using Voronoi polygons in a reference volume element (RVE) as a representation of the spherulite micro-structure. This model was extended in [9] by considering the amorphous and crystalline fractions within the spherulites with a laminar composite model. Bédoui et al. [10] compared two different types of the representation of the lamellar microstructure of the spherulites: the laminar composite model and crystallites embedded in an amorphous matrix. Recently, Glüge et al. [11] estimated the effective Young’s modulus depending on the crystallinity. For this they introduced a two step homogenization approach: A local laminate stiffness is subjected to orientation averaging.

Laschet et al. took a first step in order to consider the specific structure of the lamellae within the spherulites [12,13]. Earlier, Charbon and Swaminarayan just interpreted the resulting directions of the front tracking method as the directions of the lamellae [2]. In Laschet et al. [12,13] some characteristics of the lamellae structure are modeled (as crystallization degree, branching, and lamella twist) within a RVE. This RVE is used to get a constant stiffness matrix in order to represent the material behavior. However, the development of this structure is not simulated, but the structure is predefined in the RVE. Additionally, as in any of the other models, only a distinction between the amorphous and crystalline phase is made. However, there exists an intermediate phase between the crystalline phase and the amorphous phase called the rigid amorphous phase (see [14]). The rigid amorphous phase is only considered by Glüge et al. [11]. However, it governs together with the crystalline phase the general mechanical response (see Jabbari et al. [15] and Yeh et al. [16]).

In this paper the mechanical properties in terms of the stiffness of semi-crystalline polymers are predicted under consideration of the detailed lamellar structure within the spherulites. For this purpose, structure simulations will be presented at two different scales: On the one hand, at the nanoscale, the growth of the lamellae and the formation of spherulites due to branching of the lamellae are simulated. For this, in Section 2 the four-phase model of polymer crystallization and the derived simulation model at the microscale are introduced. Thereby the rigid amorphous phase is considered, which has a high influence on the material behavior (see [17]). On the other hand, at the microscale, the growth of the spherulites is simulated without considering the detailed lamellar structure within. Two evolution equations are used to describe the nucleation and the growth of the spherulites, which are presented in Section 3. In Section 4 the determination of the mechanical properties under consideration of both the micro- and the nanoscale, is described.

## 2. Nanoscale Structure Simulation

### 2.1. Four-Phase Model of Polymer Crystallization

**Figure 1:**
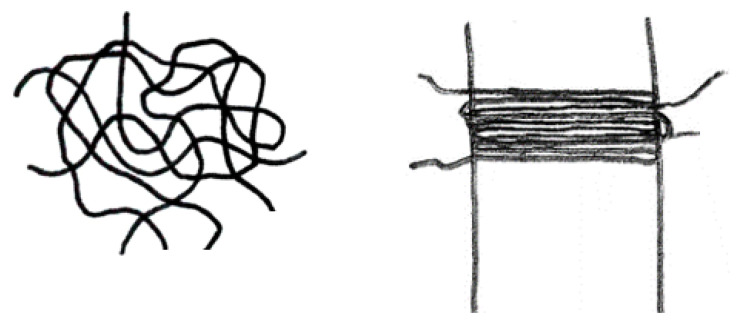
Schematic representations of the amorphous fraction (**left**) and the crystalline fraction (**right**). The vertical lines are indicating the border of the lamella.

For the phase conversion at the nanoscale, the multiphase model according to Strobl [18,19] is one of the most accepted models in the literature. This model considers the amorphous, mesomorphic, and crystalline fractions. The basis is the concept of Keller [1] which states that the polymer chains in the amorphous fraction (AF) are entangled and in the crystalline fraction (CF) they are regularly folded (see Figure 1). According to Strobl [18,19], the mesomorphic fraction (MF) occurs in the temporal development from the amorphous to the crystalline fraction. In this phase, the polymer chains are already folded, but the physical bonds are still weak, so that this phase initially remains flexible and mobile. Thus, the mesomorphic fraction is a temporal transition phase, which only occurs during the crystallization process.

According to Di Lorenzo and Righetti [20] the general amorphous fraction has to be divided into a mobile and a rigid fraction in order to be able to describe the structural characteristics of almost all semi-crystalline polymers—also due to the fact that a perfect regular folding, as assumed by Strobl [18,19], does not exist in reality. The rigid amorphous fraction (RAF) is an intermediate phase that contains segments of molecular chains that continue into both the CF and the mobile amorphous fraction (MAF). These chain segments are more stretched and less entangled than in the MAF and have a considerably reduced mobility. Rastogi et al. [17] experimentally proved the influence of the RAF on the mechanical properties.

**Figure 2:**
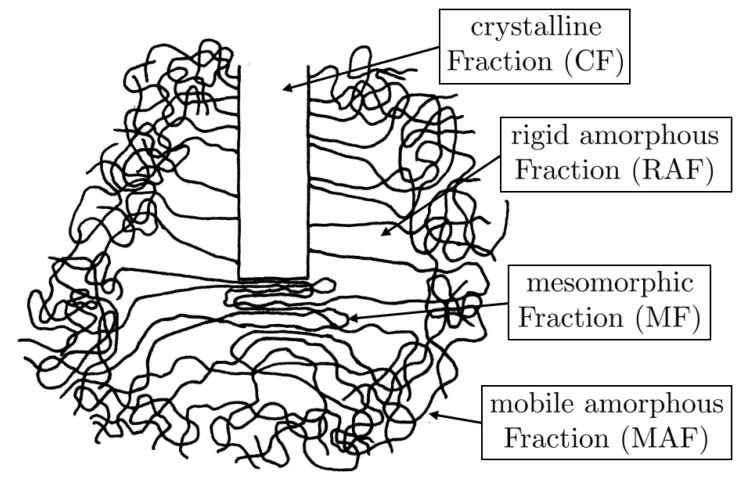
Schematic representation of the lateral growth of a crystallite to the lamella according to the four phase model, based on the model of Strobl [18].

Due to this, the four-phase model of polymer crystallization has been developed (see also Horn et al. [21]). Based on Strobl’s model, it additionally considers the RAF. As indicated in the schematic representation in Figure 2, the starting point is a lamella nucleus of crystalline fraction in an entirely mobile amorphous polymer, where the polymer chains are entangled and form coils. In order to achieve lateral growth of the lamella, the polymer chains have to partially dissolve their entanglements and fold within the MF. To realize the folding of the polymer chains, chain length is required. This leads to stretched polymer chains around the MF, which form the RAF. Within the MF, the long-range order of the polymer chains with respect to each other is continuously increased until the MF turns into the CF and the lamella is growing. In the course of time, the polymer chains in the RAF relax through molecular movements, so that the RAF partially re-converts into the MAF.

In order to depict the several fractions more clearly, in Figure 2 MAF, RAF, and MF are separated from each other. Contrary to this, in the polymer these fractions will overlap. However, as explained above, MF and RAF are always emerging together. Additionally, due to the stretched state in the RAF, the mobility of the polymer chains is significantly reduced. As a consequence, the RAF obstructs the formation of the CF.

### 2.2. Simulation

For the implementation of the crystallization model, a 2D-cellular automaton was used. In each cell the same local rules were defined, describing the development of the phase components. The rules (and thus the development of the phase components) depend only on the state of the cell and the states of the neighboring cells in the previous time step. For this purpose, the fractions of MAF $P_{ij}^{a}$ , MF $P_{ij}^{m}$ , RAF $P_{ij}^{r}$ , and CF $P_{ij}^{c}$ are defined on each cell ij . For the mathematical calculations, the cell is combined with its neighboring cells in the set N . The summation of a state variable of all cells of the set N is expressed with $\sum_{N}$ . For the sake of clarity, the index ij (indicating the cell position) is omitted in the following.

Three physically motivated rules have proven to be sufficient to represent the complex aspects of crystallization: A diffusion rule, a conversion rule, and a branching rule. The **diffusion rule** represents the mobility of the polymer chains, which are mapped as continuous phases in the model. It is based on the anisotropic second Fick’s law for diffusing media, which is discretized by the Central Difference Method. The MAF and the RAF are regarded as diffusible, in CF and MF diffusion is suppressed.

**Figure 3:**
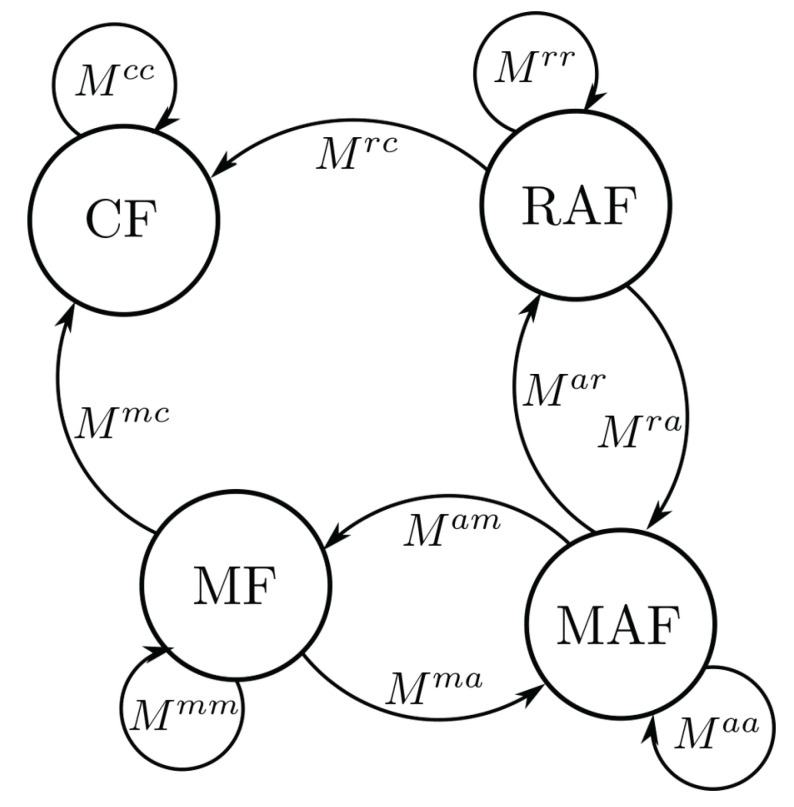
Schematic depiction of the Markov chain. Each arrow depicts a possible phase conversion and the corresponding entries of the Markov matrix are displayed next to the arrows.

The phase conversion between different phases is performed by the local **conversion rule**, whereby a conversion of certain phases is excluded (e.g., CF to MAF), since only the crystallization and not the melting is considered. In programming, the conversion was implemented by means of a Markov chain (see Figure 3), which ensures the independence of the order of the individual phase conversions. The corresponding Markov matrix on the cell ij is specified by

(1) $$\left[\begin{array}{c} M \end{array}\right]=\left[\begin{array}{cccc} 1-M^{am}-M^{ar} & M^{am} & M^{ar} & 0\\ M^{ma} & 1-M^{ma}-M^{mc} & 0 & M^{mc}\\ M^{ra} & 0 & 1-M^{ra}-M^{rc} & M^{rc}\\ 0 & 0 & 0 & 1 \end{array}\right].\;$$

The indices indicate the phase conversion specified by the coefficient. Therein, the assignment is MAF *a*, MF *m*, RAF *r*, and CF *c*. So, $M^{am}$ is the coefficient for the conversion from MAF *a* to MF *m*. All possible paths of the conversion are also shown in the Markov chain in Figure 3. A matrix multiplication with the former phase distributions and the Markov matrix leads to the new phase distributions:

(2) Pat+1Pmt+1Prt+1Pct+1=MtTPatPmtPrtPct

Thus, the Markov matrix must be recalculated for each time step. In the following equations the rules for the calculation of the Markov matrix are given. Each component depends only on the phases of the current time step, so that an explicit calculation of the Markov matrix is possible. For the sake of clarity, the index indicating the time step *t* is omitted below.

The phase conversion from MAF to MF and RAF is encouraged by the surrounding MF and CF, which is taken into account by the quantity

(3) $$c=\min\left\{2\left[\sum_{N}\left(P^{m}+P^{c}\right)\right],1.0\right\}$$

In addition, the distance of the cell to the front of the nearest lamella *r* has to be considered. Additionally, the before mentioned simultaneous emerging of MF and RAF as well as the more long-range impact of the formation of the RAF (considered by $\sum_{N}c$ ) has to be taken into account. This results in

(4) $$M^{am}=\frac{c}{r}p_{1}$$

(5) $$M^{ar}=\frac{c}{r}\left(p_{2}-p_{1}\right)+\frac{1-c}{r}p_{3}\sum_{N}c$$

Additionally, the further phase conversion from MF to CF is favored by the surrounding MF and CF:

(6) $$M^{mc}=\frac{c}{r}p_{4}$$

When converting from RAF to CF, in addition to the supporting surrounding MF and CF it must be considered, that the surrounding RAF will hinder the conversion:

(7) $$M^{rc}=\frac{c}{r}p_{5}\left(1-\sum_{N}P^{r}\right)$$

The reconversion from MF or RAF to MAF is encouraged by the surrounding MAF, which is modeled using the equations

(8) $$M^{ma}=\left(1-\frac{M^{mc}}{p_{4}}\right)p_{8}\sum_{N}P^{a}$$

and

(9) $$M^{ra}=\left(1-\frac{M^{rc}}{p_{5}}\right)\langle \sum_{N}P^{a}-p_{6}\rangle p_{7}.$$

For $M^{ra}$ it is assumed that a certain amount of relaxed polymer chains (MAF) must be present before the stretched chains (RAF) are able to relax. $p_{1}$ to $p_{8}$ are model parameters and $\langle \;\cdot \;\rangle$ are the Macauley brackets.

**Figure 4:**
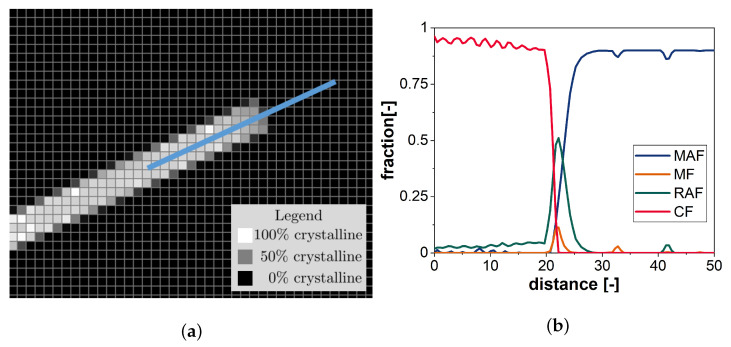
Simulation result for the growth of a lamella. (**a**) The crystalline phase of a lamella growing on both sides. The blue line marks the evaluation path along which the distribution of the phase fractions is evaluated in (**b**).

In addition to this deterministic approach, each phase conversion has a random component in order to take into account conversions due to submicroscopic fluctuations in density or temperature. As depicted in Figure 4, the combination of diffusion and conversion rule alone is sufficient to reproduce the lateral growth of the crystalline phase creating a lamella in the modeling space of the cellular automaton. The grayscale cell filling indicates the crystalline portion of the respective cell (black: 0%, white: 100%). The fraction of the individual phases determined from the simulation area along a path crossing the lamella front (see path in Figure 4a) is depicted in Figure 4b. The phase distribution clearly shows the difference between the lamella (CF content approx. 90%) and the environment. Furthermore, it provides information about the phase distribution in the lamella front as the local main conversion site of all phases in particular. The other phases occurring within the lamella in addition to the crystalline phase are interpreted as defects within the crystallite, and so the increase of crystallinity in CF due to the improvement of the crystal is also visible in the simulation results.

The **branching rule** implements the possible branching of the lamellae and is thus the basis for the development of a spherulitic superstructure. A branch is generated next to an already grown lamella, when there is sufficient MAF for the growth of a new lamella $\left(\sum_{N}P^{a}>p_{9}\right)$ and the RAF does not prevent the new formation of another lamella $\left(\sum_{N}P^{r}<p_{10}\right)$ . Taking Figure 2 into account it becomes clear that due to the RAF a branch can only occur at a certain distance behind the lamella front.

**Figure 5:**
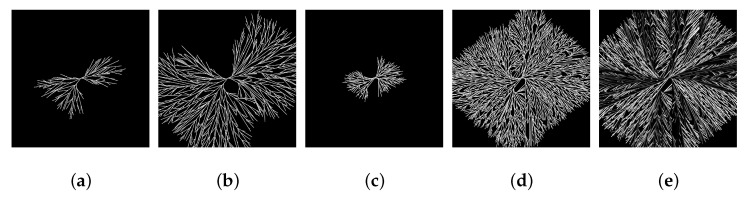
(**a**–**d**): Simulation results for the growth of a single spherulite at different time steps with a resulting degree of crystallization of (**a**,**b**): 25% and (**c**,**d**): 38% (**e**): angle-dependent depiction of a simulated spherulite.

As mentioned before, with these three rules the temporal formation of a spherulitic structure can be simulated. Figure 5a,b shows two points in time in the formation of a spherulite with a 25% degree of crystallization, while Figure 5c,d shows two points in time in the formation of a spherulite with a 38% degree of crystallization. An angle-dependent depiction of the crystalline phase, which imitates the birefringent effect in polarization microscopy, also shows the formation of a Maltese cross typical for spherulites (Figure 5e). So in its entirety, the simulation model leads to a realistic representation of the lamellar crystallization up to the temporal formation of a spherulitic structure. It includes consideration of the complex phase composition of MAF, MF, RAF, and CF. By using a variation of model parameters, different resulting degrees of crystallization can be reached.

In order to obtain characteristic values about the temperature-dependent crystallization of semi-crystalline thermoplastics, calorimetric investigations using polybutylene terephthalate (PBT) were performed. To examine the largest possible temperature range of crystallization, Fast Scanning Calorimetry (FSC) was used. In particular the Flash DSC 1 (Mettler Toledo) in combination with the UFS 1 sensor-chip was used as an analysis device. This is especially characterized by the scope of applicability of extremely high heating and cooling rates up to 40,000 K / s or 4000 K / s , respectively. Thus, despite the very high crystallization rates of semi-crystalline polymers, the specific investigation of isothermal crystallization was possible. A detailed description of the technical implementation of Fast Scanning Calorimetry can be found in [22], for example.

**Figure 6:**
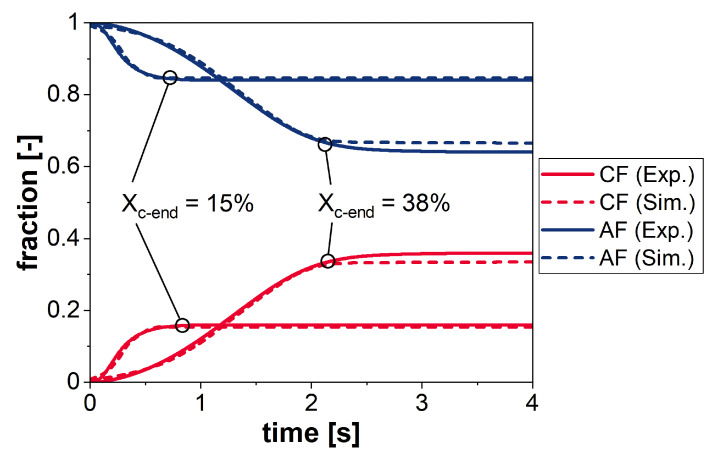
Comparison of the temporal evolution of the crystalline phase (CF) and the amorphous fraction (AF) between simulation and experiment for two different final degrees of crystallization. The phases MAF, MF, and RAF were combined to give the amorphous fraction (AF).

Thus, isothermal crystallization experiments at several temperatures were performed with Flash DSC 1 as validation for the simulation results. Figure 6 depicts the course of the phase transition for two exemplary temperatures, resulting in different degrees of crystallinity. Due to experimental limitations, only the crystalline phase fraction and the amorphous fraction were depicted. So, the amorphous fraction combines the remaining phases of the model (MAF and RAF). In order to compare these experimental data with the simulation results, the parameters $p_{1}$ to $p_{10}$ were identified for the different degrees of crystallinity. For both resulting crystallinities, the temporal development of experiment and simulation coincides and the derived crystallization rate or kinetics can be satisfactorily represented.

## 3. Microscale Structure Simulation

At this simulation scale instead of simulating the development of lamellae, general evolution equations for nucleation and growth rate of spherulites were used. In nucleation, homogeneous and heterogeneous nucleation are distinguished. Homogeneous nucleation is the beginning of crystallization due to thermally induced molecular motion of the polymer chains, while heterogeneous nucleation refers to the beginning of crystallization due to impurities, nucleation agents, or crystallites that have not been completely melted [23]. In order to describe the different nucleation mechanisms, Choe and Lee [24] derived evolution equations from the theory of phase transition kinetics by Tobin [25]:

(10) $${\dot{N}}^{\mathrm{hom}}=N_{0}\exp\left(-\frac{E_{d}}{RT}\right)\exp\left(-\frac{\Psi_{1}T_{m}^{0}}{T\left(T_{m}^{0}-T\right)}\right)\;N^{\mathrm{het}}=\frac{k}{4\pi \mu_{0}^{3}}.$$

${\dot{N}}^{\mathrm{hom}}$ is the nucleation rate of the homogeneous nucleation, which indicates the number of new nuclei per time and volume. $E_{d}$ is the activation energy of diffusion of crystallizing segments across the phase boundary, *R* the universal gas constant, $\Psi_{1}$ a constant related to the free energy of formation of a growing embryo, *T* the temperature of the melt, and $T_{m}^{0}$ is the equilibrium melting temperature of the polymer. $N_{0}$ is a material-specific constant for homogeneous nucleation. In contrast to homogeneous nucleation, in heterogeneous nucleation the number of nuclei $N^{\mathrm{het}}$ is specified directly with the material-specific parameters *k* and $\mu_{0}$ . In order to simulate the additional nucleation points due to the roughness of the mold and impurities on the mold, an additional nucleation density at the boundaries $N^{\mathrm{het}}\;\mathrm{BC}$ is used at the contact points with the environment.

In addition, the evolution equation for the growth rate of spherulites was taken from Mandelkern [26] and Cormia et al. [27]:

(11) $$\mu =\mu_{0}\exp\left(-\frac{E_{d}}{RT}\right)\exp\left(-\frac{\Psi_{2}T_{m}^{0}}{T\left(T_{m}^{0}-T\right)}\right)$$

where $\Psi_{2}$ is a constant related to the free energy of formation of a critical nucleus. With these approaches, the expression of the spherulite structure could be simulated in a 2D-cellular automaton under different boundary conditions.

In order to describe the temperature development within the simulation area the heat equation

(12) $$\frac{\partial T}{\partial t}=a\Delta T$$

with a constant thermal diffusivity *a* being used.

**Figure 7:**
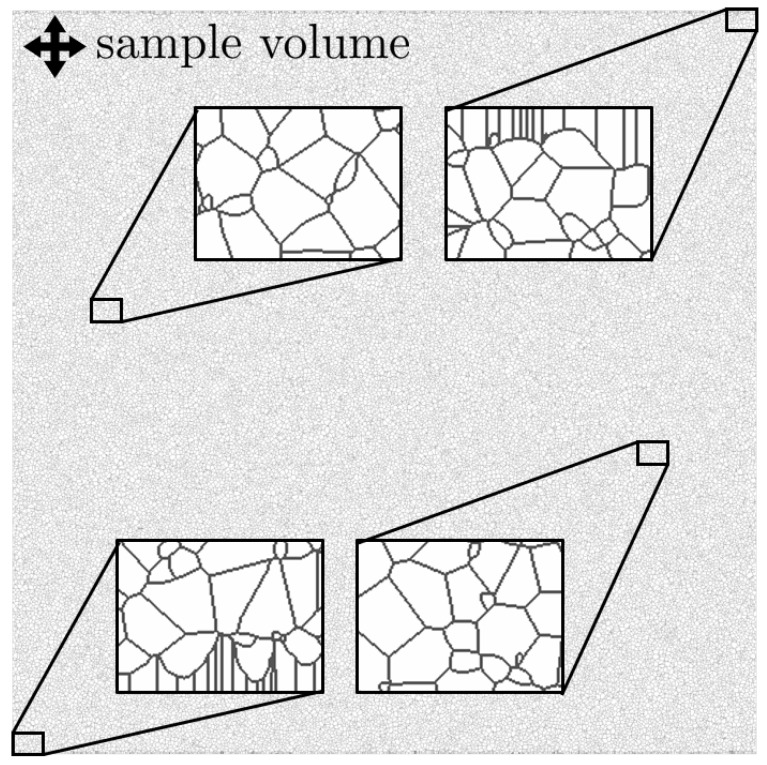
Depicted is the spherulite structure of a sample volume with four detail views. The edge structure as well as the approximately homogeneous spherulite size in the center of the simulation area can be seen.

Figure 7 depicts a simulation result for a simulation range of about $50\;{\mathrm{mm}}^{2}$ for a theoretical cooling of an initial temperature of $T_{i}=327$ ${}^{\circ }C$ and an ambient temperature of $T_{a}=27$ ${}^{\circ }C$ . Periodic boundary conditions were used for the left and right edges, while at the upper and lower edge the material is in contact with the environment. Both the almost homogeneous spherulite size inside the simulation area and the clear development of edge structures can be seen.

**Figure 8:**
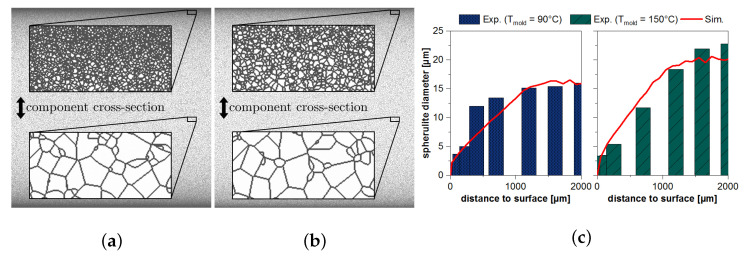
Simulated spherulitic microstructure of PBT after cooling at a mold temperature of (**a**) 90 ${}^{\circ }C$ and (**b**) 150 ${}^{\circ }C$ . (**c**) Comparison of the mean diameters of the spherulites determined by experiment and simulation.

In Figure 8a,b the simulation of the spherulitic microstructure is depicted under practical boundary conditions (mold temperature $T_{\mathrm{mold}}=90$ ${}^{\circ }C$ or $T_{\mathrm{mold}}=150$ ${}^{\circ }C$ , $T_{m}=265$ ${}^{\circ }C$ each) over the components cross-section of a 4 mm plate. It is clear that a gradient of the spherulite sizes from the parts core to the surface can be calculated. There is also a clear difference due to the change in mold temperature. In Figure 8c these exemplary calculated gradients are compared to the experimentally recorded spherulite size distributions in different component depths at different mold temperatures.

**Figure 9:**
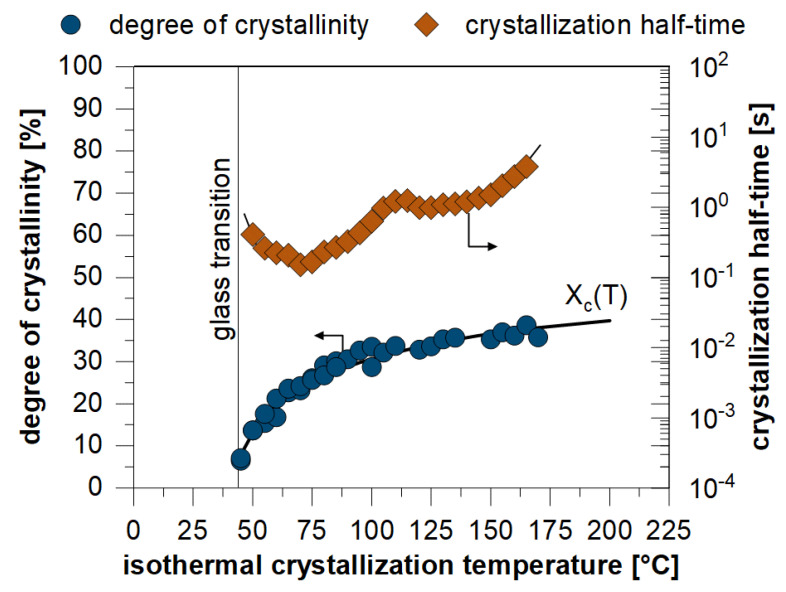
Dependence of the degree of crystallization on temperature for PBT, determined by experiments.

Through the experimental use of the FSC, the crystallization behavior of PBT could be analyzed for a large temperature range. Figure 9 shows the resulting crystallinity as well as kinetic aspects in the form of the crystallization half-time dependence on the isothermal crystallization temperature. The data points of the resulting crystallinity can be approximated by the function

(13) $$X_{c}=-6.68+0.46\left(1-\exp\left(-\frac{T}{75.92}\right)\right)+6.65\left(1-\exp\left(-\frac{T}{10.65}\right)\right)$$

By considering this dependence in the crystallization simulation, it is possible to determine not only the spherulite structure, but also the local degree of crystallization.

## 4. Predicting the Material Behavior under Consideration of the Micro- and Nanoscale

The simulation at the nanoscale provides detailed information about the structure and mechanical properties of the investigated material. However, the time for the calculation increases massively with the level of detail, so that it is not possible to calculate a complete component with the same level of detail as the nanoscale. On the other hand, the simulation at the microscale allows calculations at component size scale, but it has a much lower level of detail due to the fact that the internal structure of the spherulites is not represented. In order to combine the positive and purposeful approaches from the nanoscale simulation with the efficient calculation of the microscale, a suitable cross-scale combination was developed. For this purpose, a homogenization of the nanoscale was performed in order to predict the mechanical properties of the component, based on the structural simulations and in combination with the results of the microscale.

For this homogenization, as a first step the results of the structure simulations at the nanoscale were transferred to the FE program Abaqus to determine the mechanical properties of the structure. For this purpose a CPE4 element was assigned to each cell and the entire simulation space was designed as a 2D representative volume element (2D-RVE) with periodic boundary conditions analogous to Goldberg et al. [28]. A different Neo-Hooke material law was assigned to each phase and the crystalline phase was additionally anisotropically stiffened in order to consider the direction-dependent properties of the lamellae—see also [29,30]:

(14) $${\overset{˜}{\underset{=}{T}}}_{N}^{j}=G^{j}{\left(\mathrm{dev}\left[J_{3}^{-\frac{2}{3}}\underset{=}{C}\right]\;\cdot \;\overset{-1}{\underset{=}{C}}\right)}^{S}+K^{j}J_{3}\left(J_{3}-1\right){\underset{=}{C}}^{-1}\;\mathrm{with}\text{:}\;J_{3}=\det\left[\underset{=}{F}\right]\;j\in \left\{a,m,r,c\right\}$$

(15) $$\overset{c}{\underset{=}{\tilde{T}}}={\underset{=}{\tilde{T}}}_{N}^{c}+G_{F}\left(\mathrm{tr}\left[\underset{=}{C}\;\cdot \;\underset{=}{A}\right]-1\right)\exp\left(2{\left(\mathrm{tr}\left[\underset{=}{C}\;\cdot \;\underset{=}{A}\right]-1\right)}^{2}\right)\underset{=}{A}\;\mathrm{with}\text{:}\;\underset{=}{A}=\underline{a}\circ \underline{a}$$

where $\underline{a}$ is the direction of the lamellae, $\underset{=}{F}$ the deformation gradient, $\underset{=}{C}={\underset{=}{F}}^{T}\;\cdot \;\underset{=}{F}$ , and $\underset{=}{\tilde{T}}$ the second Piola–Kirchhoff tensor. Thus, the total stress at each cell is computed as follows, taking into account the phase distributions present at the cells:

(16) $$\underset{=}{\tilde{T}}=P^{a}{\underset{=}{\tilde{T}}}_{N}^{a}+P^{m}{\underset{=}{\tilde{T}}}_{N}^{m}+P^{r}{\underset{=}{\tilde{T}}}_{N}^{r}+P^{c}\overset{c}{\underset{=}{\tilde{T}}}$$

The material parameters at the nanoscale were determined by means of the available literature sources [31,32,33] for the parameters given in Table 1.

Thus, the mechanical properties at the nanoscale can be determined.

**Figure 10:**
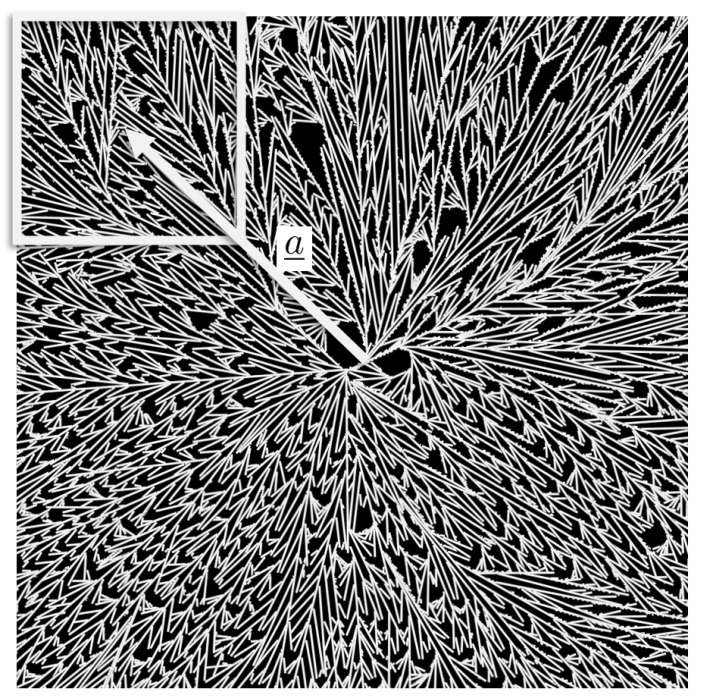
A spherulite at the nanoscale, where a subregion and the not normalized vector $\underline{a}$ from the center of the spherulite to the center of the subregion is marked.

In order to determine the material properties as a function of the degree of crystallization and of the position in the spherulite, the calculated spherulites of the nanoscale simulation were subdivided into subregions. Each of these subregions was transformed into a 2D-RVE and the mechanical properties in each of these subregions were computed, in order to be able to differentiate the local differences in anisotropy. Thereafter, the mechanical behavior of these subregions was approximated by means of a Neo-Hooke material law with anisotropic reinforcement:

(17) $$\underset{=}{\tilde{T}}={\underset{=}{\tilde{T}}}_{N}+\sum_{i=1}^{2}G_{Fi}\left(\mathrm{tr}\left[\underset{=}{C}\;\cdot \;{\underset{=}{A}}_{i}\right]-1\right)\exp\left(2{\left(\mathrm{tr}\left[\underset{=}{C}\;\cdot \;{\underset{=}{A}}_{i}\right]-1\right)}^{2}\right){\underset{=}{A}}_{i}\;\mathrm{with}\text{:}\;{\underset{=}{A}}_{i}={\underline{a}}_{i}\circ {\underline{a}}_{i}.$$

where ${\underline{a}}_{1}$ is the normalized direction from the center of the spherulite to the center of the subregion ( ${\underline{a}}_{1}=\frac{\underline{a}}{\left|\underline{a}\right|}$ , see Figure 10) and ${\underline{a}}_{2}$ is perpendicular to ${\underline{a}}_{1}$ ( ${\underline{a}}_{1}\;\cdot \;{\underline{a}}_{2}=0$ ). The material parameters of this material law were adjusted by using a material parameter optimization. Based on that, a large number of given spherulites with different phase distributions and directional characteristics have been analyzed. This analysis as well as the selection of subregions of different sizes allowed the development of a prediction method. With this method, the material parameters of the Neo-Hooke material law with anisotropic amplification as a function of the directional distribution and the degree of crystallization $X_{c}$ can be predicted. For all material parameters, a cubic ansatz was found to be suitable:

(18) $$y=aX_{c}^{3}+bX_{c}^{2}+cX_{c}+d$$

**Figure 11:**
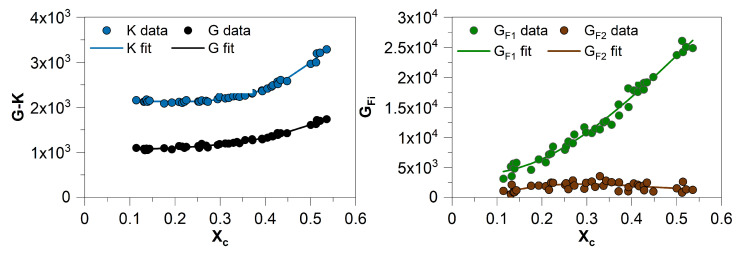
Data and associated fit for the dependence of the material parameters on the degree of crystallization.

Table 2 contains the determined parameters and Figure 11 depicts the data and the corresponding fits.

**Figure 12:**
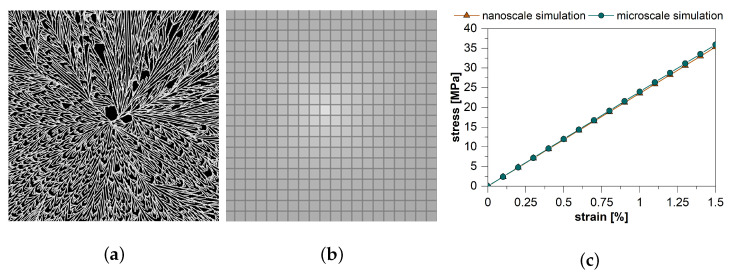
(**a**) Used section of a spherulite in the nanoscale simulation. Depicted is the crystalline fraction. (**b**) Division of the section of the spherulite in the microscale simulation. In the microscale simulation, the lamellar structure is no longer available, only the degree of crystallization and the distance as well as the direction to the nucleus of the spherulite are present on the individual cells. Depicted is the degree of crystallization, the cell edges are marked. (**c**) Calculated material response from the nanoscale simulation compared with the material response from the microscale simulation using the material parameter prediction.

In Figure 12 the application of the method for the prediction of material parameters is exemplarily shown on a section of a single spherulite. For this purpose, a spherulite from the nanoscale simulation (Figure 12a) was transferred to the FE-simulation and the material response to a given strain in the elastic range was calculated on the basis of the phase distribution (Figure 12c). Subsequently, the spherulite was simulated in the microscale simulation (Figure 12b) and by means of the prediction of the material parameters and the Neo-Hooke material law with anisotropic stiffening, the material response to the same strain was also calculated. Figure 12c compares the two material responses. It is shown that the transfer of the calculated mechanical properties from the nano- to the microscale is possible.

**Figure 13:**
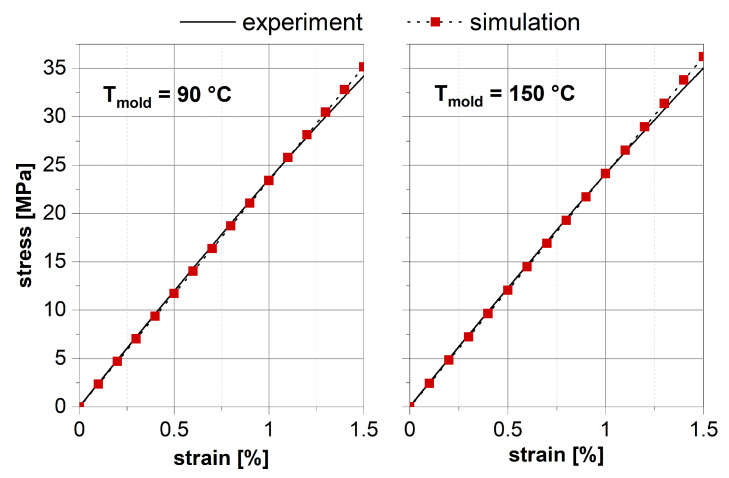
Comparison of experimentally determined and simulated stress responses to a given strain in an elastic tensile test for mold temperatures of 90 ${}^{\circ }C$ and 150 ${}^{\circ }C$ .

This approach to material parameter prediction allows the crystallization structure to be considered in a component simulation. An injection molded PBT tensile test specimen in accordance with DIN EN ISO 527 Part 1/2 was chosen as a demonstrator component. The mold temperature was varied between 90 ${}^{\circ }C$ and 150 ${}^{\circ }C$ for the production of different tension specimens. A quasi-static tensile test was then carried out with these specimens and the stress–strain curve was recorded (see Figure 13). Then, crystallization simulations were performed at the microscale with the boundary conditions which prevail in the specimen production (see Figure 8). The crystallized spherulite structure was then transferred to the finite element method (FEM) and the stress–strain curve was calculated for a tensile load. For this purpose, the Neo-Hooke material law with anisotropic reinforcement (see Equation (17)) was used. The material parameters were determined locally via the material parameter prediction according to the local degrees of crystallization calculated in the microscale simulation (see (18)).

Figure 13 shows the comparison of the experimentally measured and simulated stress responses for the two different mold temperatures. The simulation results correlate very well with the experimental data up to a strain of 1.2% , but at higher strains there is a deviation. This deviation results from the fact that the material models only consider an elastic and no viscoelastic material behavior.

## 5. Conclusions

This paper presents a possibility to determine the mechanical properties of a semi-crystalline thermoplastic simulatively, taking into account its crystalline structure. For this purpose, a crystallization simulation was carried out both at the nanoscale of the lamellae and at the microscale of the spherulites.

At the nanoscale (see Section 2) it was shown that with the presented four-phase model it is possible to depict the growth of the crystalline regions in the form of lamellae starting from a nucleation point. The temporal evolutions of the different phases of the semi-crystalline polymer determined from this model achieved good agreement with the experimentally determined curves. Subsequently, the results of the crystallization simulation were transferred to the FEM and the mechanical properties of individual spherulites and also of sections of these spherulites were determined by means of RVE homogenization.

At the microscale (see Section 3) evolution equations known from the literature were used for a crystallization simulation as well. Due to the larger scale, crystallization can be simulated in a substantially larger area. However, the simulation results do not show the individual lamellae within the spherulites anymore, but only simulate the spherulite growth. By means of an experimental study, it was possible to establish a dependence of the degree of crystallization on the local crystallization temperature of the melt. This dependence was subsequently used to calculate the local degree of crystallization in the simulation at the microscale.

In order to consider the mechanical properties of the nanoscale within the microscale, a large number of nanoscale simulations with many variations of parameters were performed. This allowed a determination of dependence of mechanical properties (in terms of parameters of a material law) on the local degree of crystallization. In addition, a prediction of material parameters based on the local degree of crystallization alone could be established (see Section 4). By transferring the results of the crystallization simulation at the microscale into the FEM, the mechanical properties of components can be predicted as a function of the microstructure. A comparison of the simulatively predicted and experimentally determined mechanical properties of two components with different spherulite structures provided very satisfactory results in the scope of elasticity.

So, as far as we know, this paper is the first to present a simulation of the lamellar structure of spherulites of semi-crystalline polymers. However, this has so far been limited to a 2D simulation space. Due to this, for example the semi-crystalline polymers with spiral growth of the lamellae cannot be simulated or can just be simulated with a systematic error. Therefore, the objective in future investigations is to extend the nanoscale as well as the microscale simulation to 3D.

In addition, the used homogenization method was applied for the first time to semi-crystalline polymers. Currently, this is limited to elastic properties. However, this method can easily be transferred to viscoelastic properties as well. If these viscoelastic properties are taken into account, a prediction up to the initiation of plastic flow is possible. Due to this, it is planned to include them in the following work.

## Figures and Tables

**Table 1:** The material parameters used to determine the mechanical properties at the nanoscale.

*G*	*K*	$G^{a}$	$K^{a}$	$G^{m}$	$K^{m}$	$G^{r}$	$K^{r}$	$G^{c}$	$K^{c}$	$G_{F}$
337.47 MPa	737.45 MPa	0.5G	0.5K	*G*	*K*	*G*	*K*	5G	5K	$10\frac{9GK}{3K+G}$

**Table 2:** Fit parameters for the cubic ansatz (Equation (18)) for PBT.

*y*	*a* [MPa]	*b* [MPa]	*c* [MPa]	*d* [MPa]
*G*	8254	−3448	898	995
*K*	22,882	−10,867	1547	2071
$G_{F1}$	−100,699	164,423	−19,201	4415
$G_{F2}$	85,642	−106,204	39,893	−2497

## Data Availability

Data available on request due to restrictions eg privacy or ethical.

## Abbreviations

The following abbreviations are used in this manuscript:

AF	Amorphous Fraction
CF	Crystalline Fraction
DSC	Differential Scanning Calorimetry
FE	Finite Element
FEM	Finite Element Method
FSC	Fast Scanning Calorimetry
MAF	Mobile Amorphous Fraction
MF	Mesomorphic Fraction
PBT	Polybutylene Therephthalate
RAF	Rigid Amorphous Fraction
RVE	Representative Volume Element
